# A Case Report on Uremic Toxins and Their Effects on Cardiac Rhythm: Understanding Junctional Ventricular Escape Rhythm in Renal Failure

**DOI:** 10.7759/cureus.43302

**Published:** 2023-08-10

**Authors:** Manjari Sharma, Mukosolu F Obi, Yash Garg, Karthik Seetharam, Hyun Joon Cho

**Affiliations:** 1 Internal Medicine, Wyckoff Heights Medical Center, Brooklyn, USA; 2 Cardiology, Mount Sinai Hospital, New York, USA

**Keywords:** cardiogenic shock, uremia, ventricular escape rhythm, junctional bradycardia, brash syndrome

## Abstract

Understanding the reasons behind junctional ventricular escape rhythm is crucial for guiding the clinical management of patients. Various factors such as acidosis, hyperkalemia, metabolic toxins, digoxin toxicity, and BRASH syndrome (comprising bradycardia, renal failure, atrioventricular (AV) nodal blockade, shock, and hyperkalemia) should be considered when dealing with a symptomatic unstable patient in a hospital. In this case, we present an example where metabolic toxins, specifically uremia, in combination with other factors, lead the patient to enter a ventricular escape rhythm, ultimately resulting in cardiogenic shock. The main objective of this case study is to illustrate how uremic metabolic acidosis contributes to AV nodal blockade, leading to a junctional ventricular escape rhythm within the context of BRASH.

## Introduction

A junctional ventricular escape rhythm is a cardiac rhythm where the origin of pacemaker activity is located in the ventricles, specifically the bundle of His. This occurs when the sinoatrial (SA) node and atrioventricular (AV) nodes are blocked or conduct slower than the automaticity of the bundle. Consequently, the bundle of His takes over and controls the heart's rhythm and rate due to its inherent pacemaker activity. Typically, the ventricular conduction rate in junctional rhythm ranges from 20 to 40 bpm. There are various factors that can trigger a junctional ventricular escape rhythm, including digoxin toxicity, BRASH syndrome, hyperkalemia, acidosis, infection, medication-induced causes, and ischemic disease [1].

In this particular case, we investigate the role of uremia and metabolic acidosis as contributing factors that synergistically lead to the development of a junctional ventricular rhythm within the context of BRASH syndrome. A BRASH syndrome is a clinical syndrome characterized by the acronym representing bradycardia, renal failure, AV nodal blockade, shock, and hyperkalemia. Several studies have highlighted how patients with acute kidney injury experience impaired potassium clearance and the effects of AV nodal agents, ultimately resulting in bradyarrhythmia and shock; however, this case highlights uremia as a contributing factor [2].

## Case presentation

**Figure 1:**
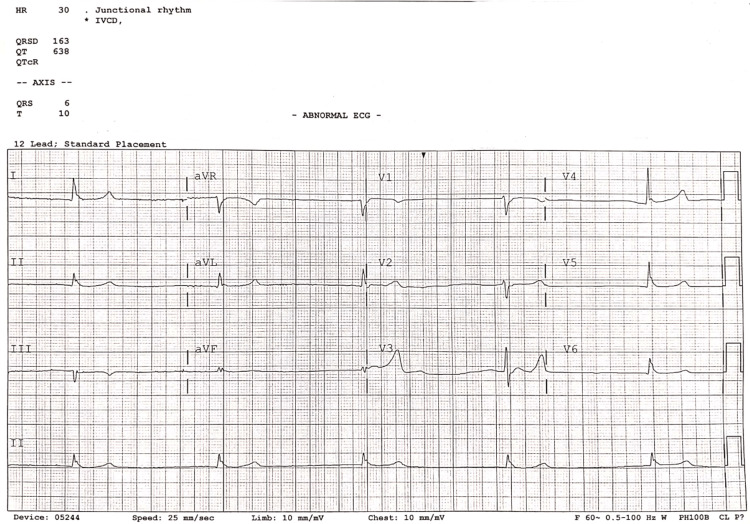
EKG showing ventricular junctional escape rhythm with no p-waves and widened depolarization of ventricles, heart rate of 30 bpm

A 68-year-old male presented to the hospital with hypotension, diaphoresis, and nausea. The patient had a past medical history of coronary artery disease with four stents, hypertension, diabetes mellitus, chronic kidney disease stage 5, and peripheral artery disease with stent placements. Medications consisted of finasteride, carvedilol, amlodipine, insulin, pantoprazole, aspirin, sodium bicarbonate, low-dose gabapentin once a day, famotidine, furosemide, lisinopril, and aspirin. During the initial evaluation, the patient was alert and oriented to time, place, and name. He was bradycardic with a low heart rate of 31 bpm. His blood pressure was measured at 76/35 mmHg, while his oxygen saturation level on a non-rebreather was saturating at 97%. EKG quickly showed a junctional rhythm (Figure 1). A few minutes later, the patient abruptly became altered and then unresponsive. He was afebrile and clinically fluid overloaded with crackles heard in the lung fields and bilateral pitting edema up to the hip. He was emergently intubated for airway protection with a Glasgow coma score of 6. Biochemistry including potassium and blood urea nitrogen, troponin, and arterial blood gas is shown in Tables 1-2. Labs showed hyperkalemia and uremia. The patient had an anion gap of 20.0, and arterial blood gas analysis showed metabolic acidosis. The bradycardia was treated with 1.4 mg of atropine and 2 grams of calcium gluconate. He was treated with furosemide 40 mg IV one-time dose which also helps in managing fluid overload as well as hyperkalemia. Additionally, the patient was given insulin 10 units to help normalize the potassium. A bicarbonate drip was initiated to restore the acid-base balance. While still in the emergency department, a dopamine drip was started in an attempt to increase the blood pressure without any improvement. Dopamine was exchanged for norepinephrine resulting in an increase in mean arterial pressure and heart rate. ICU was consulted and the patient was taken for emergent hemodialysis for severe metabolic acidosis in the setting of uremia and hyperkalemia. After the first session of hemodialysis, the patient reverted back to sinus rhythm with premature ventricular contractions. Blood pressure increased to normal range. He was extubated and transferred to the medical floor for observation.

**Table 1:** Biochemistry on admission showing hyperkalemia and uremia prior to dialysis and after dialysis

Chemistry	Values	Values After Dialysis	References
Sodium Level (mmol/L)	130	136	136-145
Potassium Level (mmol/L)	6.1	3.7	3.5-5.1
Chloride Level (mmol/L)	101	102	98-107
Bicarbonate Level (mmol/L)	9	15	21-32
Creatinine Level (mg/dl)	15.30	11.40	0.55-1.30
Blood Urea Nitrogen (mg/dl)	173	123	7-18
Glucose Level (mg/dl)	476	198	74-106
Corrected Calcium (mg/dl)	7.9	8.6	8.5-10.1
Lactic Acid Level (mmol/L)	1.6	-	0.4-2.0
Troponin Level (ng/L)	46.2	-	3.0-58.9

**Table 2:** Arterial blood gas on admission before and after dialysis pCO2: partial pressure of carbon dioxide; pO2: partial pressure of oxygen; HCO3: bicarbonate

Arterial Blood Gas	Values Prior to Dialysis	Values After Dialysis	References
pH	7.07	7.33	7.350-7.450
pCO2 (mmHg)	28	25	35.0-45.0
pO2 (mmHg)	167	207	80.0-100.0
HCO3 (mmol/L)	8.1	13.2	25.0-27.0

## Discussion

In patients with end-stage renal disease, the accumulation of uremia leads to the development of an acidic environment. This acidosis has a significant impact on the AV node, as it becomes more sensitive to lower pH levels, ultimately reducing both the conduction and contractility of the cardiac myocytes [3]. Consequently, bradycardia occurs. Additionally, electrolyte imbalances, particularly in potassium levels, also contribute to the occurrence of bradycardia. Normal potassium levels are crucial for the generation of action potentials within the cardiac myocytes. In the case at hand, the patient had been refusing hemodialysis for a week, resulting in an increase in both uremia and potassium levels. The elevated uremia triggered slow conduction in the heart, which was further exacerbated by the elevated potassium levels. This combination ultimately led to the development of a junctional rhythm and, subsequently, shock.

The presence of acidosis leads to a delay in conduction, resulting in the prolongation of the PR interval and, in severe cases, the occurrence of AV nodal blocks. Interestingly, the posterior nodal extension (also known as the slow pathway) within the AV node appears to be particularly susceptible to the effects of acid. This sensitivity intensifies the conduction delay even more. The mechanism underlying the prolongation of the PR interval is likely attributed to two primary factors. Firstly, there is a decrease in the sodium current, which plays a significant role in the conduction process. Secondly, an increase in intercellular resistance contributes to the impairment of conduction. These two mechanisms combined contribute to the prolongation of the PR interval in the presence of acidosis [3].

AV nodal disruptions can also be caused by hyperkalemia, which refers to abnormally high levels of potassium in the blood. Elevated potassium levels lead to depolarization of the resting membrane potential, resulting in an acceleration of conduction velocity. However, when potassium levels exceed 8 mmol/L, the conduction velocity decreases. This disruption in conduction can be observed on an EKG, where the action potential is characterized by the broadening and eventual disappearance of the p waves [4]. Consequently, a bradyarrhythmia-like junctional rhythm can occur. While junctional rhythms have been primarily associated with very high potassium levels ( K:8 mmol/L) or high potassium levels (K:6.5 mmol/L) with the presence of beta-blocker therapy, it has been observed that moderately elevated potassium levels, even in the absence of beta-blocker therapy, can lead to the development of junctional rhythm [5]. Renal failure or uremia, along with hyperkalemia, are among the clinical manifestations associated with a syndrome called BRASH syndrome [2]. In the presented case report, the patient arrived with hypotension and bradycardia, which occurred in the context of uremia and hyperkalemia. The EKG revealed a junctional rhythm with a heart rate of 31 bpm. Junctional rhythm occurs when independent pacemaker sites within the ventricles take control of heart rhythm in the absence of proper functioning of the SA node, atria, and AV node. The heart rate typically ranges from 20 to 40 bpm.

The BRASH syndrome has been widely discussed in various reviews, highlighting its association with acute renal failure, which hampers the clearance of AV nodal medications, and hyperkalemia, resulting in the manifestation of clinical symptoms such as bradycardia, AV nodal dysfunction, and shock [1]. However, this review sheds light on the synergistic impact of uremia, a non-selective beta blocker (carvedilol, 3.25 mg twice a day) that the patient was taking, and hyperkalemia on the conduction system. The combination of these factors caused a delay in the conduction system and a reduction in conduction velocity. Consequently, the patient developed bradycardia with a junctional rhythm, eventually leading to cardiogenic shock.

## Conclusions

To summarize, previous reviews have established a connection between the use of AV nodal blocking agents, hyperkalemia, and the development of junctional ventricular rhythm in the BRASH syndrome. However, it is vital to highlight that in this specific case, the simultaneous presence of uremia, hyperkalemia, and beta blockers played a significant role in triggering bradycardia and subsequent shock. This underscores the complex interrelationship among these contributing factors and underscores the importance of comprehending their interplay to effectively manage and treat cardiovascular complications.
